# Incidence and clinical features of ANCA-associated vasculitis before and during the COVID-19 pandemic: experience from a single-center in Northern Spain

**DOI:** 10.3389/fmed.2026.1766518

**Published:** 2026-02-06

**Authors:** Ligia Gabrie, Fabricio Benavides-Villanueva, Héctor Miguel Ulloa-Alvarado, Vanesa Calvo-Río, Iván Ferraz-Amaro, Santos Castañeda, Marcos López-Hoyos, Ricardo Blanco

**Affiliations:** 1 Department of Rheumatology, Marqués de Valdecilla University Hospital, Santander, Spain; 2 Immunopathology Group, Valdecilla Health Research Institute (IDIVAL), Santander, Spain; 3 Cantabrian Health Service, Primary Health Care, Santander, Spain; 4 Department of Rheumatology, Complejo Hospitalario Universitario de Canarias, San Cristóbal de La Laguna, Spain; 5 Department of Rheumatology, Hospital Universitario de La Princesa, IIS-Princesa, Madrid, Spain; 6 Department of Immunology, Marqués de Valdecilla University Hospital, Santander, Spain

**Keywords:** ANCA-associated vasculitis, antineutrophil cytoplasmic antibody (ANCA), COVID-19, epidemiology, pandemic, SARS-CoV-2, systemic vasculitis

## Abstract

**Background:**

A transient increase in anti-neutrophil cytoplasmic antibody (ANCA)-associated vasculitis (AAV) incidence was observed during the coronavirus disease 2019 (COVID-19) pandemic. This study aimed to assess new AAV diagnoses during the COVID-19 pandemic in a single-center cohort.

**Methods:**

We conducted a retrospective observational study of patients newly diagnosed with AAV at a tertiary care university hospital in Northern Spain between January 2019 and December 2022. Cases were classified according to the 2022 American College of Rheumatology/European Alliance of Associations for Rheumatology (ACR/EULAR) criteria. Clinical and serologic data were collected, including antibody specificity for proteinase 3 (PR3-ANCA) and myeloperoxidase (MPO-ANCA), as well as the temporal relationship to SARS-CoV-2 infection or COVID-19 vaccination.

**Results:**

A significant increase in AAV incidence was observed during the pandemic, rising from 22.4 cases per million in 2019 to 37.9 cases per million in 2021 (*p* = 0.031). Approximately 40% of patients diagnosed during this period had a recent SARS-CoV-2 infection or had been vaccinated against COVID-19 in the preceding 3 months. By 2022, AAV incidence returned to pre-pandemic levels. Contrary to initial trends, demographic and clinical characteristics remained stable; no significant differences were observed in age, gender distribution, disease severity, or organ involvement between the pre-pandemic and pandemic periods. While a numerical increase in PR3-ANCA cases was noted during the pandemic, MPO-ANCA remained the predominant subtype.

**Conclusion:**

The temporary but significant rise in AAV incidence suggests a possible temporal association with COVID-19 infection or vaccination. These findings underscore the need to conduct larger, multicentre studies to confirm these observations, investigate potential pathophysiological mechanisms, and improve clinical management approaches.

## 1 Introduction

Anti-neutrophil cytoplasmic antibodies (ANCA)-associated vasculitis (AAV) includes a group of distinct entities characterized by necrotising vasculitis of small- and medium-sized blood vessels and the presence of ANCA. These antibodies typically target myeloperoxidase (MPO-ANCA) or proteinase 3 (PR3-ANCA) proteins (1). AAV comprises three well-differentiated conditions: granulomatosis with polyangiitis (GPA), microscopic polyangiitis (MPA), and eosinophilic granulomatosis with polyangiitis (EGPA) (1, 2). The presence of PR3-ANCA is characteristic of GPA while MPO-ANCA is predominant in MPA. However, many patients present with overlap syndromes (e.g., MPO-ANCA–positive GPA) (3). Therefore, in patients suspected of having AAV, it is recommended to test for both PR3 and MPO-ANCA using a high-quality antigen-specific assay as a first-line diagnostic method (3, 4).

The incidence of AAV has increased over time, likely due to improvements in ANCA testing, new disease classification criteria, and heightened clinical suspicion (1, 5). The etiology of AAV involves genetic, environmental, and infectious factors (2, 6), with ANCA and neutrophils playing a central role. However, the mechanisms leading to ANCA production remain unclear. Neutrophils are critical in the pathogenesis of AAV. They are primed by inflammatory cytokines leading to increased expression of PR3 and MPO granules (3, 7). These granules may be recognized by anti-PR3 or anti-MPO antibodies, resulting in neutrophil degranulation (3, 8). Priming may be triggered by various factors, including drug exposure, activation of the alternative complement pathway, or several infections (3, 9).

Coronavirus disease 2019 (COVID-19) is caused by Severe Acute Respiratory Syndrome Coronavirus 2 (SARS-CoV-2) (10). Several serious complications, including various autoimmune diseases, have been observed in these patients (10–13). Both SARS-CoV-2 infection and COVID vaccination have been reported as potential triggers of AAV (10, 13–16), although it remains unclear whether this represents a true association or merely a coincidental finding. The impact of the COVID-19 pandemic on AAV incidence also remains uncertain. Clustering of AAV cases following lockdown periods has been reported, possibly due to delayed presentation or diagnostic activity. However, a single-center study in Austria described clustering of AAV cases in the absence of diagnostic delay, and one center observed clustering only in AAV patients with milder symptoms. Nonetheless, post-lockdown clustering has not been consistently observed (2). While several case reports and small series have linked COVID-19 infection and vaccination to AAV onset, large, single-center epidemiological studies investigating the impact on annual incidence and subtype distribution remain scarce.

Taking all these considerations into account, our study aimed to evaluate, in a cohort of patients from a single tertiary university hospital, the following two objectives in the context of the COVID-19 pandemic: (a) the frequency of positive ANCA tests; and (b) the frequency and characterization of AAV syndromes, analyzed across two different periods of time: pre-COVID-19 and during the COVID-19 pandemic.

## 2 Methods

### 2.1 Study design and population

We conducted a single-center observational study at a university hospital in the region of Cantabria, Northern Spain, with a reference population of approximately 580,000 inhabitants. Case confirmation was based on laboratory identification of positive ANCA tests. Therefore, patients with AAV who remained persistently ANCA-negative were not included in this analysis. The medical records of all patients with newly detected ANCA positivity in 2019, 2021, and 2022 were reviewed to identify incident cases. These patients were divided into two groups: ANCA-positive with AAV and ANCA-positive without AAV.

Patient classification into AAV subtypes (GPA, MPA, and EGPA) was established according to the 2022 American College of Rheumatology/European Alliance of Associations for Rheumatology (ACR/EULAR) classification criteria (17–19). Patients who did not meet the criteria for AAV were classified as unclassified AAV.

We analyzed two different periods: one pre-pandemic year (2019) and two pandemic years (2021–2022). Data from 2020 were excluded to avoid bias related to diagnostic delays during pandemic lockdowns. However, to provide context regarding a potential diagnostic backlog, it should be noted that 48 new ANCA-positive patients were recorded during 2020, with 17 of them receiving a diagnosis of AAV.

Given the introduction of COVID-19 vaccines in 2021 and the evolving epidemiological trends, the years 2021 and 2022 were analyzed both together and independently.

### 2.2 Data collection

Clinical and laboratory data were obtained from electronic medical records and entered into an anonymised database, including: presence of fever, constitutional symptoms (asthenia, anorexia, weight loss), and specific organ involvement (ear, nose, and throat, pulmonary, renal, cutaneous, neurological, and cardiovascular). Disease activity and prognosis at the time of diagnosis were assessed using the Birmingham Vasculitis Activity Score (BVAS) (20) and the Five Factor Score (FFS) (21). Treatment approaches (induction and maintenance therapies such as glucocorticoids, rituximab, cyclophosphamide) were also recorded. Additionally, a follow-up evaluation was performed 1 year after the initial positive ANCA results. All data were verified independently to minimize transcription errors.

The primary outcome of the study was to compare the annual incidence rate of AAV between the pre-pandemic (2019) and pandemic (2021–2022) periods. Secondary outcomes included the comparison of clinical phenotypes, disease severity (BVAS/FFS), and the temporal association with SARS-CoV-2 infection or vaccination.

### 2.3 Clinical definitions, laboratory data and outcomes

Fever was defined as a body temperature above 38 °C. Constitutional symptoms were defined as asthenia, anorexia, and unintentional weight loss >5% within the 6 months prior to diagnosis.

The erythrocyte sedimentation rate (ESR) and serum C-reactive protein (CRP) were assessed as the most common biomarkers of inflammation. An elevated CRP was defined as a value > 0.5 mg/dl. An ESR >20 mm/1st hour in men or >25 mm/1st hour in women was considered abnormal.

Outcomes were assessed at 1-year follow-up in both time periods. The EULAR consensus definitions for disease activity in AAV were used to evaluate effectiveness. Remission was defined as the absence of typical signs, symptoms, or features of active AAV (4). Clinical remission specifically referred to a BVAS (version 3) score of zero (20).

### 2.4 ANCA determination

Anti-MPO and anti-PR3 antibodies were determined using a chemiluminescence assay (BIO-FLASH, Inova Diagnostics, San Diego, CA, USA), following the manufacturer's protocol. A value of 20 chemiluminescent units (CU) was established as the cut-off point to consider positive results for ANCA. The measuring range for anti-MPO antibodies was 3.2–739.8 CU, and for anti-PR3 antibodies it was 2.3–3,285.3 CU. Indirect immunofluorescence (IIF) testing for ANCA was not performed (22).

### 2.5 Statistical analysis

Statistical analysis was performed using IBM SPSS Statistics, version 20.0 (SPSS Inc., Chicago, IL, USA). Shapiro–Wilk test was used to assess normality of distribution. Continuous variables are presented as mean ± standard deviation (SD) or median and interquartile range (IQR), with 95% confidence intervals (95% CI) when applicable. Group comparisons for continuous variables were made using Student's *t*-test or Mann–Whitney *U* test, depending on distribution. When comparing more than two groups, one-way ANOVA or Kruskal–Wallis test was applied, as appropriate. Changes in laboratory parameters at 1 year were analyzed using the Wilcoxon signed-rank test.

Categorical variables are presented as counts and percentages. Comparisons were made using the Chi-squared test or Fisher's exact test, as appropriate. A *p*-value ≤ 0.05 was considered statistically significant.

Annual incidence rates were calculated by dividing the number of newly diagnosed AAV cases by the population at risk for each year. Population data were obtained from the Cantabrian Health Service (https://www.scsalud.es/memoria-d.g-salud-p%C3%BAblica-2022) and the Spanish National Statistics Institute (INE; https://www.ine.es). All data were anonymised prior to analysis to ensure patient confidentiality.

### 2.6 Ethical approval

This study was approved by the Cantabria Clinical Research Ethics Committee (internal code: 2020.167) and conducted in accordance with the principles of the Helsinki Declaration, as revised in Fortaleza, Brazil (2013).

## 3 Results

### 3.1 New positive ANCA tests and new AAV diagnoses

**Figure 1:**
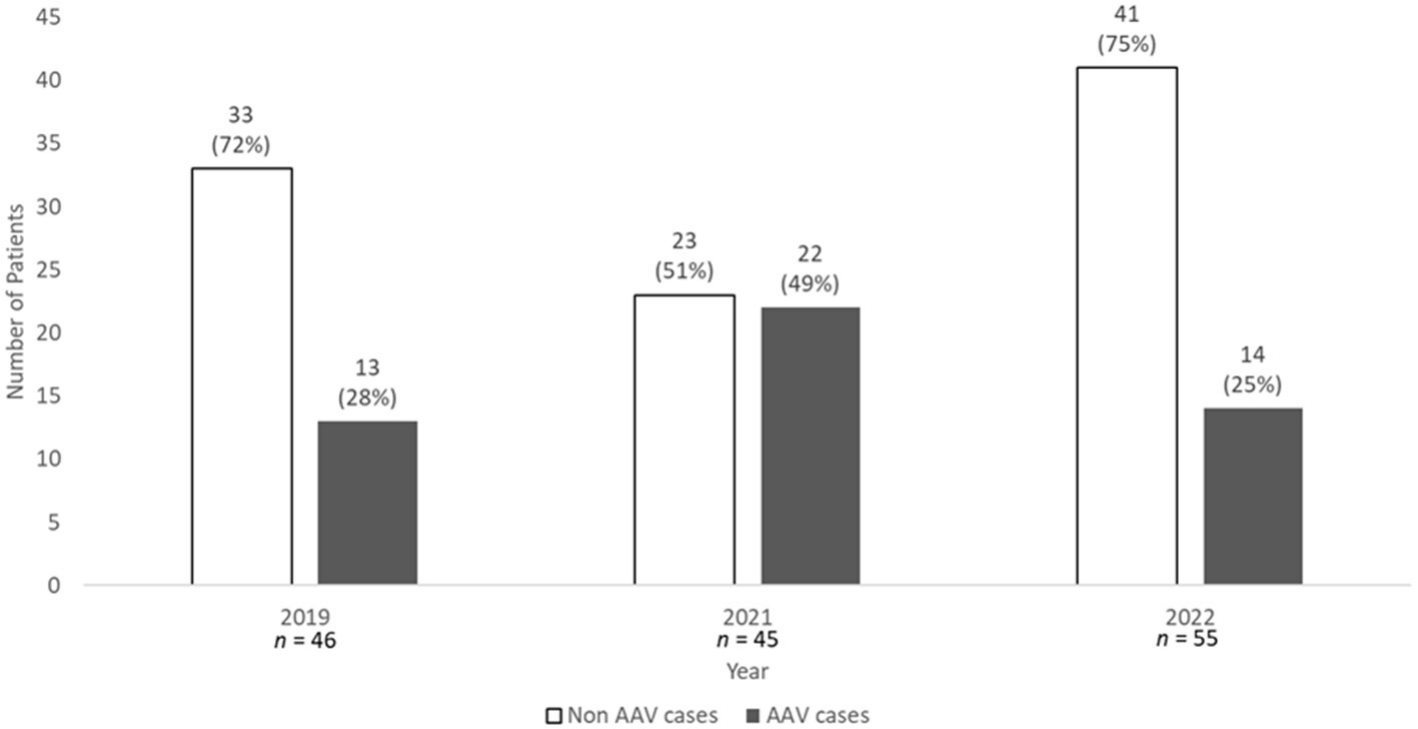
Comparison of patients with newly positive ANCA test results in the pre-COVID-19 and COVID-19 periods, and the subsequent development of AAV. AAV, ANCA-associated vasculitis; ANCA, anti-neutrophil cytoplasmic antibodies; COVID-19, coronavirus disease 2019; *n*, number. Statistics: *p*-Value according to Pearson's Chi-Square test = 0.031.

In our center, ANCA determinations were performed in 1,290, 1,434, and 1,687 patients during 2019, 2021, and 2022, respectively. Among these, the percentage of patients with newly positive ANCA tests remained stable: 3.6% (46 patients) in 2019, 3.1% (45 patients) in 2021, and 3.3% (55 patients) in 2022. However, the proportion of these cases diagnosed with new-onset AAV varied. Specifically, 13 (28%), 22 (49%), and 14 (25%) of the newly ANCA-positive cases were diagnosed with AAV in 2019, 2021, and 2022, respectively (Figure 1). Consequently, the proportion of new AAV diagnoses was significantly higher in 2021 (*p* = 0.031). This corresponds to a marked increase in AAV incidence from 22.4 cases per million (95% CI: 10.3–36.2) in 2019 to 37.9 cases per million (95% CI: 22.4–55.2) in 2021, before returning to pre-pandemic levels in 2022.

The non-AAV ANCA-positive group comprised a heterogeneous population with immune-mediated or chronic conditions. The most frequent diagnoses included inflammatory bowel disease, systemic autoimmune diseases (e.g., rheumatoid arthritis, systemic lupus erythematosus), infectious endocarditis, and cases of cocaine-induced ANCA. Other comorbidities included interstitial lung disease and hematologic malignancies.

### 3.2 Demographic features and ANCA specificity of new AAV

In total, 49 new cases of AAV occurred during the 3 years included in the period of study. The main clinical features of AAV cases are summarized in Table 1.

**Table 1:** General characteristics, AAV subtypes, and treatment of patients with newly diagnosed AAV during the pre-COVID-19 (2019) and COVID-19 (2021–2022) periods.

**Characteristic**	**Pre-COVID-19**	**COVID-19**			***p*** **-values**	
	**2019 (*****n*** = **13)**	**2021 (*****n*** = **22)**	**2022 (*****n*** = **14)**	**Overall, 2021 and 2022 (*****n*** = **36)**	**Year-by-year comparisons**	**Pre-COVID vs. COVID 19**
Age (years), mean ± SD	67 ± 17	67 ± 13	61 ± 17	65 ± 15	0.467^a^	0.650^b^
Male/female, *n* (% male)	6/7 (46)	11/11 (50)	7/7 (50)	18/18 (50)	0.972^c^	0.57^c^
ANCA titres (CU), median [IQR]	207 [162–718]	159 [48–664]	475.5 [91.9–739.8]	208.5 [55.9–721]	0.524^d^	0.468^e^
**ANCA-test specificity**, ***n*** **(%)**						
MPO-ANCA	12 (92)	14 (63)	10 (71)	24 (67)	0.426^f^	0.190^f^
PR3-ANCA	1 (8)	7 (32)	3 (21)	10 (28)		
Both	0	1 (5)	1 (7)	2 (5)		
**AAV type**, ***n*** **(%)**						
MPA	10 (77)	11 (50)	8 (57)	19 (53)	0.554^f^	0.437^f^
GPA	2 (15)	9 (41)	3 (21)	12 (33)		
EGPA	1 (8)	1 (4.5)	2 (14)	3 (8)		
Unclassified	0	1 (4.5)	1 (7)	2 (4)		
CRP (mg/dl), median [IQR]	6.9 [0.4–13]	5.2 [0.9–10.8]	4.8 [1.2–15.6]	5.2 [1.1–11]	0.594^d^	0.666^e^
ESR, mean ± SD	37 ± 28	42 ± 37.5	62.5 ± 37	50 ± 38	0.161^d^	0.335^e^
BVAS, median [IQR]	12 [6.5–15.5]	12.5 [6–16.5]	12.5 [11.3–16]	12.5 [6.8–16]	0.922^d^	0.855^e^
FFS, median [IQR]	3 [0.5–3]	2 [1–2.3]	2 [1–3]	2 [1–3]	0.791^d^	0.496^e^
**Treatment**, ***n*** **(%)**						
Glucocorticoids	5 (38)	9 (40)	12 (86)	21 (58)	0.015^c^	0.218^c^
IVIG	0	3 (14)	2 (14)	5 (14)	0.365^c^	0.306^g^
Plasmapheresis	1 (8)	0	1 (7)	1 (3)	0.427^c^	0.464^g^
Cyclophosphamide	4 (31)	3 (14)	6 (43)	9 (25)	0.142^c^	0.723^g^
Rituximab	4 (31)	9 (50)	4 (29)	13 (36)	0.706^c^	1^g^
Mepolizumab	1 (8)	1 (4.5)	2 (14)	3 (8)	0.580^c^	1^g^
Certolizumab	0	0	1 (7)	1 (3)	0.279^c^	1^g^
Mycophenolate mofetil	0	5 (23)	1 (7)	6 (17)	0.111^c^	0.175^g^
Azathioprine	3 (23)	1 (4.5)	2 (14)	3 (8)	0.261^c^	0.321^g^
Methotrexate	1 (8)	3 (14)	2 (14)	5 (14)	0.842^c^	1^g^

AAV, anti-neutrophil cytoplasmic antibodies (ANCA)-associated vasculitis; ANCA, anti-neutrophil cytoplasmic antibodies; BVAS, Birmingham Vasculitis Activity Score; COVID-19, coronavirus disease 2019; CRP, C-reactive protein; CU, chemiluminescent units; EGPA, eosinophilic granulomatosis with polyangiitis; ESR, erythrocyte sedimentation rate; FFS, five-factors score; GPA, granulomatosis with polyangiitis; IVIG, intravenous non-specific human immunoglobulins; IQR, interquartile range; mg, milligram; MPA, microscopic polyangiitis; MPO-ANCA, ANCA specific for myeloperoxidase; n, number; PR3-ANCA, ANCA specific for proteinase 3; SD, standard deviation.

Statistics: ^a^*p*-values calculated using ANOVA.

^b^*p*-values calculated using Student's t-test.

^c^*p*-values calculated using Pearson's Chi-Square test. For glucocorticoids, post-hoc analysis revealed a significant increase in 2022 compared to 2019 and 2021.

^d^*p*-values calculated using the independent-samples Kruskal-Wallis test.

^e^*p*-values calculated using the Mann-Whitney U test.

^f^*p*-values calculated using Pearson's Chi-Square test by Monte Carlo simulation.

^g^*p*-values calculated using Fisher's exact test.

The mean age at diagnosis was comparable between periods: 67 ± 17 years in the pre-COVID-19 period and 65 ± 15 years in the COVID-19 period (*p* = 0.650). Gender distribution was also similar across both periods (*p* = 0.57).

Regarding ANCA specificity, MPO-ANCA was more frequent than PR3-ANCA in both periods (92 vs. 8% in pre-COVID-19; 67 vs. 28% in COVID-19). Although a numerical trend toward a higher proportion of PR3-ANCA positive cases was observed during the pandemic period, this difference did not reach statistical significance (*p* = 0.190). ANCA titres were similar across the two study periods; however, within the pandemic period, median [IQR] titres appeared higher in 2022 [475.5 CU (91.9–739.8)] compared to 2021 [159 CU (48–664)], though overall year-by-year variance was not statistically significant (*p* = 0.524).

### 3.3 New AAV in both periods: subtypes, clinical features and outcomes

#### 3.3.1 AAV subtypes and clinical features

MPA was the most frequent AAV subtype in both periods (77% pre-COVID-19 and 53% COVID-19), followed by GPA and EGPA. During the pandemic period, the proportion of GPA cases rose to 33% (compared to 15% pre-pandemic), though the distribution of AAV subtypes did not differ significantly between periods (*p* = 0.437; Table 1).

Disease severity and activity remained stable; the activity index (BVAS) and severity index (FFS) were similar regardless of the study period. Concerning clinical features, no statistically significant differences were observed in the frequency of fever, joint involvement, constitutional symptoms, or organ involvement between the pre-pandemic and pandemic periods (Table 2).

**Table 2:** New AAV cases in pre-COVID-19 (2019) and COVID-19 (2021 and 2022) periods: clinical features.

**Clinical feature**	**Pre-COVID-19**	**COVID-19**			***p*** **-values** ^*^	
	**2019 (*****n*** = **13)**	**2021 (*****n*** = **22)**	**2022 (*****n*** = **14)**	**Overall, 2021 and 2022** **(*****n*** = **36)**	**Year-by-year comparisons**	**Pre-COVID vs. COVID 19**
Fever, *n* (%)	1 (7.7)	4 (18.4)	3 (21.4)	7 (19.4)	0.7	0.663
Arthralgia or arthritis, *n* (%)	1 (7.7)	3 (13.6)	4 (28.6)	7 (19.4)	0.368	0.663
Constitutional symptoms, *n* (%)	2 (15.4)	7 (31.8)	4 (28.6)	11 (30.6)	0.656	0.467
Cutaneous and mucous membranes, *n* (%)	0	1 (4.5)	0	1 (2.8)	1	1
ENT, *n* (%)	2 (15.4)	5 (22.7)	2 (14.3)	7 (19.4)	0.814	1
Chest, *n* (%)	3 (23.1)	9 (40.9)	6 (42.9)	15 (41.7)	0.56	0.322
Cardiovascular, *n* (%)	0	3 (13.6)	1 (7.1)	4 (11.1)	0.558	0.562
Abdominal, *n* (%)	1 (7.7)	0	0	0	0.268	0.265
Renal, *n* (%)	8 (61.5)	12 (54.5)	11 (78.6)	23 (63.9)	0.334	1
Nervous system, *n* (%)	2 (15.4)	6 (27.3)	1 (7.1)	7 (19.4)	0.328	1

COVID-19, coronavirus disease 2019; ENT, ear, nose and throat; n, number.

Statistics: ^*^p-values calculated using Fisher's exact test.

#### 3.3.2 Treatment patterns

Treatment patterns showed specific shifts. Glucocorticoid use significantly increased in 2022 (86%) compared to 2021 (40%) and 2019 (38%; *p* = 0.015). Rituximab was the most frequently used immunosuppressant during the pandemic period (36%), while cyclophosphamide usage fluctuated, dropping in 2021 before rising in 2022. The use of intravenous immunoglobulins (IVIG) and plasmapheresis remained low throughout the study. Detailed figures are presented in Table 1.

#### 3.3.3 Outcomes and mortality

At the 1-year follow-up, significant reductions in ANCA titres, CRP, ESR, BVAS, and FFS were observed across the cohort (Table 3). Four patients died before the 1-year assessment: one in the pre-COVID-19 group, an 87-year-old woman with an adnexal tumor of unknown origin who developed a respiratory tract infection 1 month after AAV diagnosis, and three in the COVID-19 group. In two of the latter cases, the cause of death was not clearly documented, and SARS-CoV-2 infection could not be definitively ruled out as no specific testing was performed at the time of death. The third was a 76-year-old man with vascular epilepsy and severe cognitive impairment, who presented with significant dysphagia and recurrent bronchial aspiration. Among the remaining patients, all but one were in clinical remission at the 1-year evaluation.

**Table 3:** New AAV cases in pre-COVID-19 (2019) and COVID-19 (2021 and 2022) periods: outcomes after 1-year follow-up.

**Parameter**	**Pre-COVID-19**	**COVID-19**			***p*** **-values**	
	**2019 (*****n*** = **10)**	**2021 (*****n*** = **19)**	**2022 (*****n*** = **12)**	**Overall, 2021 and 2022 (*****n*** = **31)**	**Year-by-year comparisons**	**Pre-COVID vs. COVID 19**
ANCA titres (CU), median [IQR]	31.9 [14–270]	24 [4.3–61.5]	53.8 [7.8–153]	29.7 [6.5–80]	0.428^a^	0.592^b^
CRP (mg/dl), median [IQR]	0.4 [0.4–0.8]	0.4 [0.4–0.4]	0.45 [0.4–1.8]	0.4 [0.4–0.6]	0.119^a^	0.714^b^
ESR, mean ± SD	14.4 ± 10	17.6 ± 10	17.9 ± 14	17.7 ± 11	0.722^c^	0.419^d^
BVAS, median [IQR]	0	0	0	0	–	–
FFS, median [IQR]	2 [0–3]	2 [1–2]	1 [0.5–2.5]	2 [1–2]	0.767^a^	0.878^b^

ANCA, anti-neutrophil cytoplasmic antibodies; BVAS, Birmingham vasculitis activity score; COVID-19, coronavirus disease 2019; CRP, C-reactive protein; CU, chemiluminescent units; ESR, erythrocyte sedimentation rate; IQR, interquartile range; mg, milligram; n, number; SD, standard deviation.

Statistics: ^a^*p*-values calculated using the independent-samples Kruskal-Wallis test.

^b^*p*-values calculated using the Mann-Whitney U test.

^c^*p*-values calculated using one-way ANOVA.

^d^*p*-values calculated using Student's t-test.

### 3.4 COVID-19 infection/vaccination and AAV cases

Among all patients with newly detected ANCA positivity in 2021 and 2022 (*n* = 100), vaccination records or documented SARS-CoV-2 infections were available for 88 individuals. Of these, 13 were +ANCA prior to vaccination or infection (10 in the non-AAV group and three in the AAV group). In the remaining 75 patients who developed ANCA positivity following exposure, the median time to ANCA seroconversion was significantly shorter in the AAV group compared to the non-AAV group [70 days (IQR: 28–168) vs. 134 days (IQR: 74–202); *p* = 0.012; Table 4].

**Table 4:** Interval between the last COVID-19 vaccination or documented infection and the first positive ANCA test (days).

**Patient group**	**2021 (*n* = 28)**	**2022 (*n* = 47)**	**Both 2021 and 2022 (*n* = 75)**
AAV cases, *n*	16	13	29
Days, median [IQR]	57 [21.5–122.5]	88 [31.5–184]	70 [28–168]
Non AAV cases, *n*	12	34	46
Days, median [IQR]	134 [96.5–168]	134.5 [66–212]	134 [74–202]
*p*-Value^*^	0.007	0.360	0.012

AAV, anti-neutrophil cytoplasmic antibodies (ANCA)-associated vasculitis; COVID-19, coronavirus disease 2019; IQR, interquartile range; n, number.

Statistics: ^*^p-values calculated using the Mann-Whitney U test.

In 2021, 88% (14/16) of vaccination-associated AAV cases were diagnosed after the second dose. In 2022, the majority (10/13; 77%) followed the third dose. Combined, 52% of new AAV cases occurred after the second dose and 34% after the third dose (Table 5).

**Table 5:** Distribution of ANCA-associated vasculitis cases diagnosed in 2021 and 2022 relative to the timing of COVID-19 vaccination or infection.

**Event timing**	**2021 (*n*= 16)**	**2022 (*n* = 13)**	**Both 2021 and 2022 (*n* = 29)**
AAV cases after 1st dose, *n* (%)	2 (12)	0	2 (7)
AAV cases after 2nd dose, *n* (%)	14 (88)	1 (8)	15 (53)
AAV cases after 3rd dose, *n* (%)	0	10 (76)	10 (34)
AAV cases after 4th dose, *n* (%)	0	1 (8)	1 (3)
AAV cases after 5th dose, *n* (%)	0	0	0
AAV cases after documented COVID-19 infection, *n* (%)	0	1 (8)	1 (3)

AAV, anti-neutrophil cytoplasmic antibodies (ANCA)-associated vasculitis; COVID-19, coronavirus disease 2019; n, number.

## 4 Discussion

In our study, the frequency of new-onset +ANCA tests remained comparable between the pre-pandemic period (2019) and the pandemic years (2021–2022). However, the number of newly diagnosed AAV cases increased markedly in 2021, coinciding with peaks in SARS-CoV-2 infection rates and widespread COVID-19 vaccination, before returning to pre-pandemic levels in 2022. The observed incidence rose from 22.4 (10.3–36.2) cases per million in 2019 to 37.9 (22.4–55.2) per million in 2021. This exceeds the background incidence of approximately 20 cases per million per year previously reported in Europe and North America (28). These findings suggest that SARS-CoV-2–related immune mechanisms, particularly neutrophil priming (11), may have contributed to a transient increase in AAV incidence. This aligns with previous reports in the literature. Sekar et al. (23) reported the first case of AAV following COVID-19 vaccination, and since then, an increasing number of similar cases have been described. AAV has been associated with both COVID-19 infection and vaccination (7–11, 13–16, 24–35), supporting the hypothesis that immune activation induced by viral antigens may act as a trigger in genetically or immunologically predisposed individuals.

Despite the rise in incidence, the demographic and clinical phenotype of the patients remained largely stable. As previously reported, AAV affected males and females equally (36), and there was no evidence of increased disease activity, as measured by the BVAS, or of a poorer prognosis, based on the FFS. The mean age at diagnosis was 67 ± 17 years in the pre-pandemic period and 65 ± 15 years during the pandemic, aligning with the peak incidence range of 60–70 years described in previous studies (28). Therefore, while the local incidence was higher, the disease presented with classic demographic features, suggesting that the pandemic acted as a trigger for typical AAV rather than creating a distinct clinical entity.

Regarding ANCA specificity, MPO-ANCA remained the predominant subtype in our cohort during both periods, consistent with reports by Thammathiwat et al. (29), who observed a higher frequency of MPO-ANCA in post-vaccination cases. Although the proportion of PR3-ANCA positive cases was numerically higher during the pandemic (28 vs. 8% pre-pandemic), this difference was not statistically significant (*p* = 0.190), and should be interpreted cautiously.

The treatment of AAV in this cohort reflects evolving practices shaped by disease severity and prevailing clinical guidelines (4, 37). Glucocorticoids remain the cornerstone of treatment. We observed a statistically significant increase in glucocorticoid use in 2022 (*p* = 0.015), which may indicate treatment adaptations implemented during the later stages of the pandemic. Rituximab was the most frequently used immunosuppressive agent during the pandemic period (36%). This aligns with EULAR guidelines (4) and recent evidence suggesting rituximab can be safely maintained during the pandemic without worsening COVID-19 outcomes (38). The limited application of plasmapheresis and the use of IVIG in our cohort were consistent with management strategies for refractory cases or specific clinical scenarios.

A crucial finding of our study is the temporal relationship between immune stimulation and AAV onset. Among the 29 vaccination-associated AAV cases, the majority were diagnosed after the second (52%) or third (34%) vaccine doses, in line with previous observations (27, 28, 34). Only a minority were linked to the first (7%) or fourth (3%) doses. Furthermore, the median interval from SARS-CoV-2 exposure (infection or vaccination) to ANCA seroconversion in the AAV group was 70 days, significantly shorter than the 134 days observed in the non-AAV group (*p* = 0.012).

The underlying immunological mechanisms remain to be fully clarified. In healthy individuals, mRNA vaccine booster doses have been shown to elicit enhanced CD4+ and CD8+ T cell activation, alongside a more robust innate immune response compared to the first dose (39). This intensified immunological stimulation may play a role in priming ANCA autoantibody production. The enhanced innate response triggered by booster doses has also been implicated in the emergence of both MPO- and PR3-ANCA antibodies (30, 34, 40).

This study has limitations inherent to its design. First, the retrospective, single-center nature may restrict the generalisability of findings to other populations. Second, confirmed data on SARS-CoV-2 infection were not available for all patients, precluding a definitive causal link. Third, the relatively small sample size limited the statistical power to detect significant differences in sub-analyses, such as the shift in ANCA subtypes. Finally, the absence of precise timing for symptom onset in all cases hinders a more accurate assessment of potential temporal triggers.

In summary, we observed a transient, statistically significant increase in AAV incidence during the COVID-19 pandemic, temporally associated with SARS-CoV-2 infection and vaccination, particularly following the second and third vaccine doses. While the clinical presentation and patient demographics remained stable compared to the pre-pandemic period, the notable rise in incidence and the significantly shorter time to seroconversion in AAV patients support a role for immune activation in AAV pathogenesis. Prospective, multicentre studies are needed to validate these observations and further elucidate the underlying mechanisms.

## Data Availability

The raw data supporting the conclusions of this article will be made available by the authors, without undue reservation.
